# Risk and Protective Profile of Men Who Have Sex With Men Using Mobile Voluntary HIV Counseling and Testing: Latent Class Analysis

**DOI:** 10.2196/43394

**Published:** 2023-02-16

**Authors:** Piao-Yi Chiou, Wei-Wen Tsao, Kuan-Chia Lin, Yuan-Yuan Fang, Kuan-Yin Lin, Chia-Lin Li

**Affiliations:** 1 School of Nursing College of Medicine National Taiwan University Taipei City Taiwan; 2 Department of Nursing National Taiwan University Hospital Taipei Taiwan; 3 Research and Development Committee Taiwan AIDS Nurse Association Taipei Taiwan; 4 Community Medicine Research Center National Yang Ming Chiao Tung University Taipei Taiwan; 5 Medical Affairs Cheng Shin General Hospital Taipei Taiwan; 6 Department of Post Baccalaureate Nursing College of Medicine I-Shou University Kaohsiung Taiwan; 7 Department of Internal Medicine National Taiwan University Hospital National Taiwan University College of Medicine Taipei Taiwan; 8 Center for Neuropsychiatric Research National Health Research Institutes Taipei Taiwan

**Keywords:** HIV testing, latent class analysis, men who have sex with men, mobile health, postexposure prophylaxis (PEP), preexposure prophylaxis (PrEP), risk-taking, anal sex, sexual partners, social networking

## Abstract

**Background:**

Mobile voluntary counseling and testing (VCT) for HIV has been carried out to improve the targeting of at-risk populations and HIV case detection for men who have sex with men (MSM). However, the HIV-positive detection rate using this screening strategy has declined in recent years. This may imply unknown changes in risk-taking and protective features jointly influencing the testing results. These changing patterns in this key population remain unexplored.

**Objective:**

The aim of this study was to identify the nuanced group classification of MSM who underwent mobile VCT using latent class analysis (LCA), and to compare the difference in characteristics and testing results between subgroups.

**Methods:**

A cross-sectional research design and purposive sampling were applied between May 21, 2019, and December 31, 2019. Participants were recruited by a well-trained research assistant through social networking platforms, including the most popular instant messenger app Line, geosocial network apps dedicated to MSM, and online communities. Mobile VCT was provided to participants at an assigned time and place. Demographic characteristics and risk-taking and protective features of the MSM were collected via online questionnaires. LCA was used to identify discrete subgroups based on four risk-taking indicators—multiple sexual partners (MSP), unprotected anal intercourse (UAI), recreational drug use within the past 3 months, and history of sexually transmitted diseases—and three protective indicators—experience of postexposure prophylaxis, preexposure prophylaxis use, and regular HIV testing.

**Results:**

Overall, 1018 participants (mean age 30.17, SD 7.29 years) were included. A three-class model provided the best fit. Classes 1, 2, and 3 corresponded to the highest risk (n=175, 17.19%), highest protection (n=121, 11.89%), and low risk and low protection (n=722, 70.92%), respectively. Compared to those of class 3, class 1 participants were more likely to have MSP and UAI within the past 3 months, to be ≥40 years of age (odds ratio [OR] 2.197, 95% CI 1.357-3.558; *P*=.001), to have HIV-positive results (OR 6.47, 95% CI 2.272-18.482; *P*<.001), and a CD4 count ≤349/μL (OR 17.50, 95% CI 1.223-250.357; *P*=.04). Class 2 participants were more likely to adopt biomedical preventions and have marital experience (OR 2.55, 95% CI 1.033-6.277; *P*=.04).

**Conclusions:**

LCA helped derive a classification of risk-taking and protection subgroups among MSM who underwent mobile VCT. These results may inform policies for simplifying the prescreening assessment and more precisely recognizing those who have higher probabilities of risk-taking features but remain undiagnosed targets, including MSM engaging in MSP and UAI within the past 3 months and those ≥40 years old. These results could be applied to tailor HIV prevention and testing programs.

## Introduction

### Background

Owing to the success of antiretroviral therapy for people infected with HIV, and the promotion of HIV screening and prophylactic medications for at-risk populations [1-3], the annual incidence rate of HIV infections among adults has declined globally by 31% between 2010 and 2021 [4]. In Taiwan, an estimated 90% of people living with HIV (PLWH) know their HIV status and 93% of them have initiated antiretroviral treatment, 95% of whom have achieved viral suppression [5]. To reach the World Health Organization’s goal of eliminating HIV infection by 2030, improving the access of the remaining undiagnosed target groups to screening is a critical strategy in HIV case detection and epidemic control [6].

The remaining undiagnosed PLWH require aggressive testing measures to facilitate an early diagnosis. Mobile voluntary counseling and testing (VCT) for HIV is a novel screening method in Taiwan, carried out by disseminating recruitment messages through social networking platforms to deliver testing services to specific groups at a specific time and location [7]. Compared to an outreach model of screening based at a community station, mobile VCT is more flexible with respect to the time and location of testing; therefore, the targeting of at-risk populations and case discovery are improved in the mobile VCT model [8,9]. However, the number of newly diagnosed HIV infections in Taiwan has been declining annually since 2017 [10] and the number of people that are positive for HIV associated with mobile VCT is also decreasing [7], which implies unknown changes in HIV risk-taking patterns and the influence of protective features in people who are uninfected. A possible inference could be that people who were previously at risk of HIV may have reduced their risk-taking behaviors, including avoiding having multiple sexual partners (MSP) and having unprotected anal intercourse (UAI), and abstaining from recreational drug use; concurrently, they may have adopted higher biomedical protection measures, including regular HIV testing, and when necessary, taking postexposure prophylaxis (PEP) and preexposure prophylaxis (PrEP) [11-14]. To provide HIV testing resources to those with relatively high priority needs in a mobile VCT service, it is necessary to be able to precisely recognize those who encompass a higher probability of risk-taking features and a lower probability of protective features. However, the feature classification of MSM who participate in mobile VCT remains unknown.

Latent class analysis (LCA) is a mixture model that is used to identify latent heterogeneity driven by class membership according to responses to a set of observed variables [15]. The theoretical assumption underlying LCA is that an individual shares both latent and observed categorical variables, and membership in unobserved classes can cause or explain patterns of scores across assessment indicators [16,17]. More specifically, LCA helps to identify similar characteristics among individuals to derive subgroups. LCA has been used to discern patterns of HIV risk based on demographic characteristics [18,19] and behaviors [20,21], helping define and target distinct groups. According to the theoretical basis and hypotheses of LCA, as well as previous research findings, LCA is appropriate for analysis of unidentified patterns in MSM who receive mobile VCT.

### Objective

The aim of this study was to identify the nuanced group classification based on the risk and protective profile of MSM who accepted mobile VCT using LCA, and then to compare the characteristics and HIV testing results between subgroups to further determine groups of individuals with different patterns.

## Methods

### Study Design

A cross-sectional research design and purposive sampling were applied. Participants were recruited via social networking platforms. After completing an online questionnaire, we offered mobile VCT at a designated time and location, according to the participants’ preferences, where they could obtain a rapid HIV test.

### Participants

The inclusion criteria were age ≥20 years, being literate, self-identification as an MSM, and self-reported as participating in HIV risk-associated behavior. The exclusion criteria included no self-reported risk factors for HIV infection or being HIV-positive.

### Ethical Considerations

This study was approved by the Research Ethics Committee of National Taiwan University (201903ES024), and adhered to the regulations of the Human Subjects Research Act issued by the Ministry of Health and Welfare of Taiwan and the Personal Data Protection Act issued by the National Development Council in Taiwan. After explaining the study purpose and protocol, the participants were asked to complete an online consent form and questionnaires anonymously through a provided URL; all data were processed anonymously. Participants could withdraw at any time without providing a reason. A mask could be worn during the mobile VCT processes to protect privacy. Each participant was eligible to receive an electronic gift certificate worth US $3.27 after all procedures were completed.

### Procedure and Data Collection

The study covered the period from May 21, 2019, to December 31, 2019. Participants were recruited in the same manner as described in a previous study [7]. Social networking platforms were used, including the most popular instant messenger app Line and geosocial network apps dedicated to MSM, such as Grindr, Hornet, Jack’d, Scruff, and Blued. Online communities (such as Facebook fan pages) were used to disseminate the study information and recruit participants. Testing appointments were discussed and booked through one-on-one messaging on each recruiting platform. Mobile VCT was provided by a well-trained research assistant at a designated time and location, such as a convenience store, coffee shop, park, or fast-food restaurant, for participants who live in Taipei City and New Taipei City. Study information was provided to the participants, whose identity was verified through previous messaging records. Prior to the test, participants were asked to fill out an online consent form and questionnaire, which took about 10-15 minutes to complete. Following submission of the questionnaire, the researcher and participant would review and confirm whether there were any missing or questionable answers to ensure completeness of the questionnaire. Pretest counseling was provided and a blood drop was collected via a rapid HIV testing kit. Testing results were available immediately and recorded by the research assistant, and posttest counseling and a referral to the hospital HIV case manager to confirm testing and obtain PEP and PrEP were provided. The whole screening process took approximately 30-40 minutes. The CD4+ T-cell counts of each participant were followed and recorded by the research assistant.

### Instruments

#### Questionnaire

The online questionnaire included 18 closed-ended questions; six were focused on demographic characteristics and 12 were focused on risk-taking and protective features. Demographic characteristics of interest included age, education level, marital experience, monthly income, sexual orientation, and if participants were open about their MSM status. HIV risk-taking features included preferred sexual position; number of sexual partners within the past 3 months; frequency of condom use during the previous 10 occasions of anal intercourse within the past 3 months; experience of recreational drug use within the past 3 months, including the number of types of drugs; and history of sexually transmitted diseases (STDs; including syphilis, gonorrhea, genital herpes, chlamydia, human papillomavirus, pubic lice, and hepatitis A, B, and C). HIV-preventing features included awareness of PEP and PrEP, experience of using PEP and PrEP, experience of HIV testing, and frequency of HIV testing.

#### Rapid HIV Self-Testing Kits

The Alere Determine HIV-1/2 rapid testing kit was used to test for HIV-1/HIV-2 antibodies in the human serum or plasma. This kit is licensed by the Taiwan Food and Drug Administration, and has specificity and sensitivity values of 99.87% and 99.75%, respectively [22]. Results were obtained within approximately 15 minutes and were managed and recorded by trained staff.

### Indicator Selection for LCA

Risk-taking– and protection-related indicators were considered conditionally independent and were defined based on previous studies [11-14] and expert input; risk-taking–related indicators were defined as behaviors that directly lead to HIV infection or the features that significantly increase the risk of HIV infection, and the protection-related indicators were defined as proactive HIV screening or preventive drug administration. Three specialists, including an infectious disease physician, HIV case manager, and HIV public health scientist, provided expert input. Finally, seven indicators were selected. The four risk indicators included MSP, UAI, recreational drug use within the past 3 months, and history of STDs. The three protective indicators included experience of PEP use, experience of PrEP use, and regular HIV testing (at 3-6–month intervals when having unprotected sex). All variables were dichotomized (yes vs no).

### Statistical Analyses

All data were confirmed to be complete prior to analysis. LCA was performed using the poLCA package within R software (GitHub, Inc) to identify distinct classes based on model fit. The multiple fit statistics, including log likelihood (LL), likelihood ratio test statistic (G^2^), Akaike information criterion (AIC), Bayesian information criterion (BIC), and *χ*^2^ goodness of fit, were applied and considered prior to the decision on the model being made [17]. BIC was identified as the best reliable fit statistic model, which is an intuitive calculation method that is commonly used for model selection in univariate and multivariate logistic regression due to its simplicity, and considers the effect of a large sample size to prevent the model from being too complex as a result of high model accuracy [17,23,24]. In addition, we examined the similarity of item-response probability patterns within classes and the interpretability of class separation. Subgroup membership was assigned based on the class of each participant and the highest probability of belonging. A probability of ≥50% per item indicated that group members were more likely to possess a characteristic of interest [19]. The univariable multinomial logistic regression model was applied to determine differences in class characteristics and testing outcomes.

## Results

### Study Population

In total, 1023 participants made an appointment for mobile VCT via social networking platforms during the study period. Five (0.49%) participants did not attend the testing appointment and lost contact without a reason. A total of 1018 participants completed the questionnaire and entered the screening process. The participants’ characteristics are presented in Table 1. The participants’ median age was 30.17 (IQR 25-34) years. Most participants were in the age range of 20-29 years, had college- or university-level education, had never been married, and had a stable monthly income of approximately US $714-$1783. Most participants were MSM, and had had a come-out experience, preferred both top and bottom sex positions, had both casual partners and MSP, and frequently used condoms (7-9 times per 10 sexual encounters). Some participants self-reported using recreational drugs (116/1018, 11.39%) within the past 3 months, with the majority indicating the use of one or two substances. Approximately one-fifth had a history of STDs, including, human papillomavirus (106/222, 47.7%), syphilis (71/222, 31.98%), gonorrhea (50/222, 22.5%), hepatitis B (33/222, 14.9%), pubic lice (31/222, 13.96%), genital herpes (18/222, 8.1%), chlamydia (11/222, 4.95%), hepatitis A (11/222, 4.95%), and hepatitis C (10/222, 4.5%). Most participants were aware of PEP, although few had used it. Most participants were aware of PrEP, although again few had used it. Furthermore, most participants had experience of HIV testing, including testing every 3-6 months. Eighteen participants were newly HIV-positive; 9 of whom had a CD4 count ≥350/μL and nine of whom had a CD4 count ≤349/μL.

**Table 1:** Descriptive characteristics of the men who have sex with men using mobile voluntary HIV counseling and testing (N=1018).

Characteristic			Participants, n (%)
**Age group (years)**			
	20-29	545 (53.54)	
	30-39	361 (35.46)	
	≥40	112 (11.00)	
**Education**			
	High school or below high school	121 (11.89)	
	College or university	733 (72.00)	
	Postgraduate	164 (16.11)	
**Marital experience**			
	No	985 (96.76)	
	Yes	33 (3.24)	
**Monthly income (US $)**			
	≤713	214 (21.02)	
	714-1783	640 (62.87)	
	≥1784	164 (16.11)	
**Sexual orientation**			
	MSM^a^	844 (82.91)	
	Bisexual	174 (17.09)	
**Have come out**			
	No	165 (16.21)	
	Yes	853 (83.79)	
**Preferred sexual position**			
	Top	265 (26.03)	
	Bottom	287 (28.19)	
	Both bottom and top	466 (45.78)	
**Sexual partner^b^**			
	No sexual partner	143 (14.05)	
	A single main sexual partner	235 (23.08)	
	Casual and multiple sexual partners	640 (62.87)	
**Condom use during past 10 times of anal intercourse^b^**			
	No sexual behavior	83 (8.15)	
	Every time used (10 times)	287 (28.19)	
	Frequently used (7-9 times)	346 (33.99)	
	Occasionally used (4-6 times)	144 (14.15)	
	Rarely used (1-3 times)	99 (9.72)	
	Never used (0 times)	59 (5.80)	
**Recreational drug use^b^**			
	No	902 (88.61)	
	Yes	116 (11.39)	
**Number of recreational drugs used (n=116)^b^**			
	1–2	76 (65.5)	
	3–4	24 (20.7)	
	≥5	16 (13.8)	
**Experience of STDs^c^**			
	No	796 (78.19)	
	Yes	222 (21.81)	
**Have ever heard of and know of PEP^d^**			
	No	162 (15.91)	
	Yes	856 (84.09)	
**Experience of using PEP**			
	No	887 (87.13)	
	Yes	131 (12.87)	
**Have ever heard of and know of PrEP^e^**			
	No	121 (11.89)	
	Yes	897 (88.11)	
**Experience of using PrEP**			
	No	891 (87.52)	
	Yes	127 (12.48)	
**Experience of HIV testing**			
	No	162 (15.91)	
	Yes	856 (84.09)	
**HIV testing frequency (n=856)**			
	First time	162 (15.91)	
	Every 3-6 months	630 (61.89)	
	6 months and longer	226 (22.20)	
**HIV test result**			
	Negative	1000 (98.23)	
	Newly positive	18 (1.77)	
**CD4 count (n=18)**			
	≥350/μL	9 (50.0)	
	≤349/μL	9 (50.0)	

^a^MSM: men who have sex with men.

^b^Within the past 3 months.

^c^STD: sexually transmitted disease (including syphilis, gonorrhea, genital herpes, chlamydia, human papillomavirus, pubic lice, and hepatitis A, B, and C).

^d^PEP: postexposure prophylaxis.

^e^PrEP: preexposure prophylaxis.

### LCA Results

After entering the seven indicators selected for LCA, the outcomes of 2-6 latent classes were produced and compared. The final model was determined according to the lowest value of BIC and class separation, although the lowest values of LL, AIC, G^2^, and *χ*^2^ were inconsistent with those of BIC (Table 2). A three-class model was selected, based on fit statistics and model interpretation. Table 3 presents the probabilities of HIV risk-taking and the protective profile of the three-class model.

Classes 1, 2, and 3 represented the highest risk (n=175, 17.2%), highest protection (n=121, 11.9%), and low risk and low protection (n=722, 70.9%), respectively. Participants in class 1 had the highest risk of having MSP and UAI; they were also more likely to get tested regularly. Participants in class 2 had the highest probabilities of protective features, including experience of PEP and PrEP and regular HIV testing, whereas they had a medium risk of having MSP and UAI. Compared to those of classes 1 and 2, participants in class 3 had a lower risk of having MSP and UAI, and they tended to get regularly tested, which was considered an HIV-protection feature (Table 3).

**Table 2:** Goodness of fit indices for model selection.

Model	LL^a^	G^2^^b^	AIC^c^	BIC^d^	*χ* ^2^	*df*	Number of parameters
1-class	–3683.55	471.03	7381.11	7415.59	894.34	120	7
2-class	–3566.28	236.48	7162.56	7236.44	270.48	112	15
3-class^e^	–3524.05	152.02	7094.10	7207.39	187.85	104	23
4-class	–3509.56	123.04	7081.12	7233.81	151.71	96	31
5-class	–3498.77	101.46	7075.53	7267.63	109.18	88	39
6-class	–3488.17	80.26	7070.34	7301.84	80.26	80	47

^a^LL: log likelihood.

^b^G^2^: likelihood ratio test statistic.

^c^AIC: Akaike information criterion.

^d^BIC: Bayesian information criterion.

^e^The selected class based on the lowest BIC value.

**Table 3:** Posterior probabilities (%) for the three-class model (N=1018).^a^

Indicators			Class 1, highest risk (n=175, 17.2%)		Class 2, highest protection (n=121, 11.9%)		Class 3, low risk and low protection (n=722, 70.9%)
**Risk-taking indicators**							
	Multiple sexual partners^b^	96.5		66.3		51.1	
	Unprotected anal intercourse^b,c^	100.0		58.8		52.7	
	Experience of recreational drug use^b^	23.2		13.4		7.1	
	Experience of STDs^d^	43.8		24.6		14.0	
**Protective indicators**							
	Experience of using PEP^e^	8.4		76.3		2.8	
	Experience of using PrEP^f^	16.1		67.0		1.3	
	Regular HIV testing^g^	74.8		94.4		51.7	

^a^Probabilities greater than 50% indicate items for which members of a given class were more likely to be classified within that class.

^b^Within the past 3 months.

^c^Not using a condom every time during past 10 encounters of anal intercourse.

^d^STD: sexually transmitted disease (including syphilis, gonorrhea, genital herpes, chlamydia, human papillomavirus, pubic lice, and hepatitis A, B, and C).

^e^PEP: postexposure prophylaxis.

^f^PrEP: preexposure prophylaxis.

^g^Testing frequency between 3 and 6 months in the case of regular unprotected sex.

### Univariable Multinomial Logistic Regression Results

Class 3 was used as a reference due to the lower probability of HIV risk and protective features. Compared to those of class 3, class 1 participants were more likely to be ≥40 years old, to have an HIV-positive test result, and a CD4 count ≤349/μL. Class 2 participants were more likely to have been married (Table 4 and Table 5).

**Table 4:** Demographics and testing results of the three classes (N=1018).

Variable		Class 1^a^ (n=175, 17.19%), n (%)	Class 2^b^ (n=121, 11.89%), n (%)	Class 3^c^ (n=722, 70.92%), n (%)
**Age group (years)**				
	20-29	85 (48.6)	69 (57.1)	391 (54.2)
	30-39	58 (33.1)	39 (32.2)	264 (36.3)
	≥40	32 (18.3)	13 (10.7)	67 (9.3)
**Education**				
	High school or below high school	17 (9.7)	15 (12.4)	89 (12.3)
	College or university	127 (72.6)	84 (69.4)	522 (72.3)
	Postgraduate	31 (17.1)	22 (18.2)	111 (15.4)
**Marital experience**				
	No	166 (94.9)	114 (94.2)	705 (97.6)
	Yes	9 (5.1)	7 (5.8)	17 (2.4)
**Monthly income (US $)**				
	≤713	42 (24.0)	20 (16.5)	152 (21.1)
	714-1783	103 (58.9)	82 (76.8)	544 (63.0)
	≥1784	30 (17.1)	19 (15.7)	115 (15.9)
**Sexual orientation**				
	MSM^d^	142 (81.1)	104 (86.0)	598 (82.8)
	Bisexual	33 (18.9)	17 (14.0)	124 (17.2)
**Have come out**				
	No	26 (14.9)	21 (17.4)	118 (16.3)
	Yes	149 (85.1)	100 (82.6)	604 (83.7)
**Preferred sexual position**				
	Top	37 (21.1)	43 (28.1)	194 (26.9)
	Bottom	53 (30.3)	38 (31.4)	196 (27.1)
	Both bottom and top	85 (48.6)	49 (40.5)	332 (46.0)
**HIV testing result**				
	Negative	166 (94.9)	118 (97.5)	716 (99.2)
	Positive	9 (5.1)	3 (2.5)	6 (0.8)
**CD4 count (n=18)**				
	≥350/μL	2 (22.2)	2 (66.7)	5 (83.3)
	≤349/μL	7 (77.8)	1 (33.3)	1 (16.7)

^a^Class 1: highest risk.

^b^Class 2: highest protection.

^c^Class 3: low risk and low protection.

^d^MSM: men who have sex with men.

**Table 5:** Comparison of classes according to demographics and testing results (N=1018).

Variable		Class 1 versus Class 3 (highest risk vs low risk and low protection)		Class 2 versus Class 3 (highest protection vs low risk and low protection)	
		OR^a^ (95% CI)	*P* value	OR (95% CI)	*P* value
**Age group (years)**					
	20-29 (reference)	—^b^	—	—	—
	30-39	1.01 (0.699-1.461)	.96	0.837 (0.549-1.277)	.41
	≥40	2.197 (1.357-3.558)	.001	1.100 (0.576-2.099)	.77
**Education**					
	High school or below high school (reference)	—	—	—	—
	College or university	1.274 (0.732-2.216)	.39	0.955 (0.527-1.729)	.88
	Postgraduate	1.462 (0.760-2.812)	.26	1.176 (0.576-2.399)	.66
**Marital experience**					
	No (reference)	—	—	—	—
	Yes	2.248 (0.985-5.133)	.06	2.546 (1.033-6.277)	.04
**Monthly income (US $)**					
	≤713 (reference)	—	—	—	—
	714-1783	0.819 (0.547-1.226)	.33	1.370 (0.813-2.309)	.24
	≥1784	0.944 (0.557-1.600)	.83	1.256 (0.641-22.461)	.51
**Sexual orientation**					
	MSM^c^ (reference)	—	—	—	—
	Bisexual	1.121 (0.733-1.715)	.60	0.788 (0.465-1.364)	.40
**Have come out**					
	No (reference)	—	—	—	—
	Yes	1.120 (0.706-1.775)	.63	0.930 (0.559-1.549)	.72
**Preferred sexual position**					
	Top (reference)	—	—	—	—
	Bottom	1.418 (0.891-2.256)	.14	1.106 (0.669-1.830)	.69
	Both bottom and top	1.324 (0.878-2.053)	.17	0.842 (0.525-1.350)	.48
**HIV testing result**					
	Negative	—	—	—	—
	Positive	6.470 (2.272-18.428)	<.001	3.034 (0.749-12.297)	.12
**CD4 count (n=18)**					
	≥350/μL	—	—	—	—
	≤349/μL	17.5 (1.223-250.357)	.04	2.5 (0.100-62.605)	.58

^a^OR: odds ratio.

^b^Not applicable.

^c^MSM: men who have sex with men.

## Discussion

### Principal Results

LCA was used to derive three subgroups, based on best fit statistics and model interpretation, of MSM who underwent mobile VCT via social networking platforms. Two risk-taking indicators (having MSP and UAI) and three protective indicators (experience of PEP, experience of PrEP, and regular HIV testing) had probabilities >50% and were used to determine discrete classes in the final model. The profile of the highest risk group was class 1. The three classes were heterogeneous in age and marital history. These findings may help to identify subgroups suitable for targeted interventions and HIV testing.

MSP and UAI were two risk indicators that exceeded 50% in all three groups. Previous studies have confirmed that having experience of MSP and UAI can be one of the predictive factors for HIV-positivity rates of MSM [25,26]. The results of this study provide additional information; that is, the participants who were >40 years old were more likely to be grouped in class 1 and were at an increased risk of HIV and late diagnosis. Higher probabilities of MSP and UAI and age >40 years concurrently could be the first risk assessment items for setting priorities of those who need to be tested, especially when mobile VCT represents a competitive resource and needs to be applied precisely for those in the highest risk group. This result echoes a previous discussion that, compared to younger-aged MSM, middle-aged (40-50 years) adults are still at high risk for HIV infection [27]. The emergence of dating apps has enabled older MSM to connect with a different generation and to participate in condomless sexual activity [28]. Furthermore, feelings of loneliness and fatigue associated with HIV prevention may increase the risk of unprotected sexual encounters and the number of sexual partners among older MSM, thereby increasing the infection risk [29,30]. Additionally, the signs and symptoms of early HIV infection, including persistent influenza-like symptoms, herpes zoster, a single episode of bacterial pneumonia, thrombocytopenia, and lymphocytopenia, may be attributable to diseases of aging, and these people and their caregivers may neglect their risk for HIV and delay HIV testing; therefore, older adults are at increased risk of late diagnosis [31].

The other two risk indicators, drug use within the past 3 months and lifetime STDs, did not exceed a probability of 50% in the three subgroups. There are several possible reasons to explain this finding. Class 1 participants had a drug use probability of 23.2%, which was higher than those in the other classes and was lower than the probability of 77.3% from a previous report based on an online survey in a single Chinese city [32]. This probability was also lower than that previously reported (30%) in a pen-and-paper investigation, without any intervention, from 13 European cities [33]. This difference may be because the previous two studies investigated the experience of recreational drug use in a lifetime, which is longer than the past 3 months considered in this study. In addition, all participants in this study underwent counseling and testing; thus, the self-reported rate of drug use was likely an underestimate. That is, participants may be less likely to report drug use in face-to-face interactions than in online channels and paper questionnaires for fear of judgment and legal consequences. The probability of lifetime STDs (43.8%) in class 1 was higher than those in the other two classes, was comparable to that (43.3%) previously reported in a cross-sectional survey of American urban MSM undergoing a physical examination and STD testing [34], and was higher than that (13.0%) quoted in a study based on self-reports in a Chinese city [35]. Self-reporting of STDs may be limited by the feelings of shame and recall bias [36,37]. However, in this study, compared to the low-risk group, MSM in the highest risk group were more likely to disclose a history of STDs. These participants may believe that STDs are curable and have expected a medical referral for diagnosis and treatment through this consultation. These results suggest that in addition to MSP and UAI, lifetime STDs, especially a history of human papillomavirus, syphilis, and gonorrhea, and recreational drug use within the past 3 months could be considered as the minor risk-taking assessment items for arranging the priority for HIV testing.

The probabilities of the three protective indicators exceeded 50% in class 2 and were higher than those in the other two groups. It is noteworthy that the highest risk group, class 1, engaged in regular testing as the only protective measure, and the probability of taking PrEP and PEP was lower than that in class 2. Although this study did not investigate the reasons that hinder the use of PEP and PrEP in the highest risk group, previous studies have revealed that barriers include the lack of awareness of HIV risk behaviors, side effects and high cost of PrEP, the compliance difficulties and stigma of using PrEP [38,39], and low access to or tolerability of PEP [40]. Furthermore, age and income may have hindered the uptake of PrEP and PEP in this study. The proportion of participants aged ≥40 years in class 1 was 7.6% higher than that of class 2 and the proportion having a monthly income of ≤US $713 in class 1 was 7.5% higher than that of class 2. Older MSM may have a poorer economic status and were less likely to be aware, or have an inappropriate understanding of, PrEP than MSM ≤30 years old, which made them less likely to use it [41,42]. Improving HIV prevention may involve providing counseling on PEP and PrEP use during HIV testing for high-risk targets, including basic knowledge, side effect management, using reminders to ensure compliance, and providing financial and social support that increases the likelihood of preventive medication use [43].

Compared to those of class 3, class 2 participants were more likely to have been married. Studies have shown that approximately half of Chinese MSM intend to marry women and most of them expect to engage in extramarital homosexual relationships [44]. MSM who had recent UAI with unknown partners declared PrEP acquisition intentions [45]. Our findings still require further analysis to understand whether having experience of marriage could facilitate the adoption of HIV-prevention behaviors by MSM to protect their spouses.

Class 3 was the largest group and had lower risk and lower protective features than the other two classes. Over half of the participants in class 3 perceived themselves at risk of HIV infection and regularly undertook convenient, free, and anonymous HIV screening as a protective measure. Thus, HIV testing seems to be the best way to reach those who meet the characteristics of class 3. Accordingly, screening staff should continually reinforce this group of HIV risk-reducing behaviors and provide more protective options to promote the best preventive effect.

### Limitations

This study had some limitations. First, convenient sampling was used and participants were recruited mainly in northern Taiwan, thereby reducing the generalizability of the findings and representativeness of the sample. Second, this research was conducted before the COVID-19 outbreak, and thus our results may not be inferenced and applied to the COVID-19 pandemic, since the pattern of risky and protective behaviors could have changed during that period. Third, self-reported data are likely subject to bias, which may affect the validity of these findings. Fourth, this study was cross-sectional, precluding discussions about causality. Fifth, this research did not consider the factors that may affect the acceptance of mobile VCT of MSM with regard to social and environmental aspects, such as avoidance of screening due to discriminatory attitudes of health care providers. In follow-up research, indicators of more dimensions could be added to promote a more comprehensive analysis.

### Conclusions

MSM who underwent mobile VCT via social networking platforms exhibited different risk and protection profiles. LCA helped to derive a classification based on risk-taking and protective indicators. These results may inform policies for simplifying the prescreening assessment and precisely recognizing those who have higher risk-taking features but remain undiagnosed targets, including MSM engaging in MSP and UAI within the past 3 months and those ≥40 years old. Longitudinal studies could be conducted to evaluate the efficacy of this approach.

## Abbreviations

AIC: Akaike information criterion
BIC: Bayesian information criterion
G^2^: likelihood ratio test statistic
LCA: latent class analysis
LL: log likelihood
MSM: men who have sex with men
MSP: multiple sexual partners
OR: odds ratio
PEP: postexposure prophylaxis
PLWH: people living with HIV
PrEP: pre-exposure prophylaxis
STD: sexually transmitted disease
UAI: unprotected anal intercourse
VCT: voluntary counseling and testing
